# Changes in Properties of Soy Protein Isolate Edible Films Stored at Different Temperatures: Studies on Water and Glycerol Migration

**DOI:** 10.3390/foods10081797

**Published:** 2021-08-04

**Authors:** Hong Zhang, Lechuan Wang, Hanyu Li, Yujie Chi, Huajiang Zhang, Ning Xia, Yanqiu Ma, Longwei Jiang, Xiaonan Zhang

**Affiliations:** College of Food Science, Northeast Agricultural University, Harbin 150030, China; hongzz666@163.com (H.Z.); lechuanwang666@163.com (L.W.); lihanyu1004@126.com (H.L.); yjchi@126.com (Y.C.); xianing1981@126.com (N.X.); mayanqiu908@163.com (Y.M.); jianglw@neau.edu.cn (L.J.); xiaonanzhang@neau.edu.cn (X.Z.)

**Keywords:** SPI films, glycerol migration, water loss, storage temperature, film properties

## Abstract

Plasticizers and the water migration of edible protein films during storage can result in changes in film properties, while specific changing processes need to be further explored. In this study, glycerol-plasticized soy protein isolate (SPI) films were stored at 25 °C, 4 °C, and −18 °C for 6 weeks (relative humidity (RH), 40–50%). The glycerol migration was monitored by the glycerol migration rate and differential scanning calorimetry (DSC). Water content, low-field nuclear magnetic resonance (LF-NMR), and thermogravimetric analysis (TGA) were used to analyze the water state. The results showed that significant pores and cracks were observed after storage at 25 °C. The proportion of bound water gradually increased, and the glycerol migration rate also reached 1.3% and 0.7% at 25 °C and 4 °C, respectively. The results proved that increasing the storage temperature accelerated the loss of water and glycerol, and decreased the mechanical properties of the SPI film.

## 1. Introduction

Storage stability of packaging materials is an important indicator for evaluating packaging properties. Most packaging materials deteriorate in quality when exposed to a certain or special external environment for a long time [1,2], leading to a lower protection ability against the external environment on foods [3,4] and, thus, limiting its applications.

Physical and chemical aspects are the important factors affecting polymeric packaging properties and are mainly reflected by protein aggregation, the migration of plasticizers, or small-molecule-component diffusion [5]. Plasticizer migration occurs naturally during storage, but the process is difficult to observe and control [6]. Among numerous polymers, side chains of functional structures make proteins better film-forming materials [7]. This special advantage makes the protein films possess excellent packaging properties in the food preservation field. However, protein films are extremely sensitive to storage conditions, especially temperature [8].

Soy protein isolate (SPI) has been widely studied due to its good film-forming property [9]. Its protein content can reach more than 90%, with low cost and biodegradable superiorities [10], while strong interactions between SPI molecules lead to serious disadvantages in the film-forming process [11]. Pure SPI film without plasticizer is extremely brittle, resulting in less effective applications, mainly because the bonds formed between SPI protein chains are hydrophobic and hydrogen bonds, intermolecular disulfide bonding, and electrostatic forces [12]. For reducing the strong molecular interactions, different types of plasticizers were added to the SPI films [13].

As a food-grade plasticizer, glycerol is added to edible protein films constantly due to its ability to decrease protein inter-chain interactions while increasing the mobility of the protein chains. The mechanical properties of protein films were also enhanced by glycerol addition [14]. Studies have found that water in films can also act as a plasticizer due to its strong liquidity [12], and it can work together with other plasticizers to improve the films’ packaging properties by increasing the flexibility, dispensability, and extensibility [2]. Glycerol can enter into the β-sheet of soy protein, with hydrogen bonds weakening in this part. The hydrogen bonds that exist in β-sheets (C=O···H–N–) are then replaced by hydrogen bonds between SPI and glycerol (C=O···H–O–), which results in the increase in mobility of the SPI protein chain [15].

Due to the large surface area, glycerol migration and the effect on the films cannot be ignored [16]. The protein-rich and glycerol-rich regions in the interior of glycerol-plasticized SPI films caused heterogeneity [17]. This may be the key factor that causes glycerol migration during storage. In addition to the internal reasons, the thermodynamic effect can also lead to the migration of glycerol molecules to the films’ surface [13].

The purpose of this work was to study the changes in physical properties of the SPI films caused by the migration of glycerol and water at different storage temperatures (25 °C, 4 °C, and −18 °C). It has been found that storage temperatures can affect the rate of glycerol migration and water loss. The microstates of films after 6 weeks of storage were analyzed using ATR-FTIR and SEM, confirming that the migration of plasticizers can lead to different effects on its structure. This study explored the microscopic, mechanical, and optical properties of SPI films under different storage temperatures, and laid the foundation for mastering and slowing down the migration of plasticizers in SPI films.

## 2. Materials and Methods

### 2.1. Materials

Soy protein isolate with 93% protein content was purchased by Gushen Biotechnology Co. (Shandong, China). Glycerol and glycol were obtained from Tianli Chemical Reagent Co. (Tianjin, China). Sodium periodate and sodium formate were obtained from Macklin Biochemical Technology Co. (Shanghai, China).

### 2.2. Preparation of SPI Films

SPI films were prepared by the casting method. Glycerol (2.0 g) was accurately weighed and then dissolved with 100 mL of deionized water. SPI (3.0 g) was then added and mixed to dissolve by placing the solution at 60 °C, and it was then homogenized for 2.0 h. After filtering through double-layer gauze, the film-forming solution was poured into a silica plate (15 × 15 cm) after cooling to room temperature, and it was then dried at 50 °C for 8.0 h via a drying oven. The SPI protein films were then peeled off after forming.

### 2.3. Storage Conditions

Three common temperatures for food storage were chosen for this study. The SPI films were stored at 25 °C (room temperature), 4 °C (refrigeration temperature), −18 °C (freezing temperature) for 6 weeks under controlled relative humidity at 40–50%. The SPI films used to determine glycerol migration were weighed accurately and stored in different paper envelopes, and then stored for 6 weeks, and corresponding indexes were measured once a week.

### 2.4. Thickness

To measure the thickness, 10 points on the SPI film were randomly selected. The average value obtained was considered to be the thickness.

### 2.5. Swelling Ratio

The swelling ratio (SW) describes the water absorption capacity [18]. The SPI films were poured and soaked for 20 min with 25 mL of deionized water after being weighed. They were then removed, and the water on their surface was dried with filter paper and weighed. The values of S were determined by triplicate according to Equation (1), where *m_s_* and *m_i_* represent the weight of the SPI film after and before being swollen, respectively.

(1) $$S=\left(m_{s}-m_{i}\right)/m_{i}\times 100$$

### 2.6. Glycerol Migration Rate

The glycerol migration rate of SPI films during storage at different temperatures was determined by the method of Zhang et al. [19]. The SPI films were weighed after being stored in paper envelopes, and the stored paper envelopes were then soaked with 150 mL of distilled water, shaken for 4 h, and then drained and removed. The pH was adjusted to 7.8–8.0 with 0.05 mol/L of NaOH solution. Then, a 5.0 mL sodium periodate solution (60 g/L) was added and mixed evenly before being placed in the dark at 25 °C for 30 min. Next, 5.0 mL of glycol blended with deionized water 1:1 was added. After 20 min, 5.0 mL of sodium formate solution (1.0 mol/L) was added under the same conditions, and the pH was then adjusted to 7.8–8.0 (0.1 mol/L NaOH). The paper envelope without SPI films was used for the blank. The glycerol mobility formula was as follows (2):

(2) $$\mathrm{Glycerol}\;\mathrm{migration}\;\mathrm{rate}\left(\%\right)=\frac{(V_{1}-V_{2})\times C\times 0.0921\times 100}{m}$$

where *V*_1_ is the volume (mL) of NaOH standard solution consumed by the test sample; *V*_2_ is the volume (mL) of NaOH standard solution consumed by the blank experiment; *C* is the concentration of NaOH standard solution/(mol/L); 0.0921 is the molar mass of glycerol/(g/mmol); and *m* is the mass/(g) of SPI films.

### 2.7. Water Content and Distribution

SPI films were cut into 6.0 × 5.5 cm and then dried at 105 °C for 24 h after being weighed. They were then weighed again to record the loss in mass that was removed from SPI films, to calculate the water content.

The water distribution of SPI films during storage was determined using low-field nuclear magnetic resonance (LF-NMR) [20]. The water distribution was evaluated by analyzing the transversal relaxation time (T_2_) changes. Measurement conditions: the LF-NMR analysis instrument had a magnetic field strength of 0.47 T, a proton resonance frequency of 20 MHz, and a measurement temperature of 30 °C, and the SPI film rolls were tightly packed and placed into a chromatography vial, directly placed into a 25 mm diameter NMR tube, and subsequently immediately placed into a Bruker NMR instrument for analysis. Measurement parameters: Sixteen scans with a total of 3000 echoes were acquired over a time interval of 2.0 s. The raw data obtained were analyzed using the Carr–Purcell–Meiboom–Gill pulse sequence and CONTIN algorithms to obtain T_2_ relaxation times, and the water distribution was recorded.

### 2.8. ATR-FTIR Analysis

Fourier-transform infrared spectroscopy in attenuated total reflectance mode (ATR-FTIR) (Nicolet is 50 spectrometer) was used to analyze SPI film samples by using a ZnSe crystal with an incidence angle of 45 grades, and all spectra were collected as an average of 32 scans recorded at 4 cm^−1^ resolution, using air as the background [21].

### 2.9. Thermogravimetric Analysis (TGA)

TGA measurement was undertaken using the TGA Q5000 instrument. About 4.0 mg of SPI films were analyzed from 30 °C to 800 °C (10 °C/min) under an N_2_ atmosphere (20 mL/min).

### 2.10. Differential Scanning Calorimetry (DSC)

DSC was tested by using a thermal analyzer (TA Instruments, New Castle, DE, USA). SPI films (3–6 mg) were sealed in an aluminum pan and heated at a rate of 10 °C min^−1^ from 30 to 200 °C under an N_2_ atmosphere.

### 2.11. Transmittance of the Films

The transmittance was measured by a UV spectrophotometer (UV-Vis 2550, Shimadzu, Kyoto, Japan). The SPI films were cut into the same width as the quartz cuvette (1.0 × 5.0 cm), placed in the cuvette, and then scanned in the wavelength range of 200–800 nm, using air as the reference. The transmittance of SPI films was determined using Equation (3) [22]:

(3) A=lg(1/T)

where A is the absorbance value and *T* is the transmittance of SPI films.

### 2.12. SEM

The microstructure of SPI films at different storage times was determined using a SU 8010 scanning electron microscope (SEM) (Hitachi, Tokyo, Japan), and film samples were dried in dry glassware at room temperature. Samples were cut into a rectangular form (4.0 × 5.0 mm). Then, samples were adhered using double-sided adhesive tape, and one side of the SPI film was warped (2.0 mm) and coated with a thin layer of gold for 15 min in vacuum.

### 2.13. Water Vapor Permeability (WVP)

The WVP of SPI films was measured gravimetrically using the cup method described in a previous study with slight modifications [18]. SPI films were cut into 30 mm diameter round shapes to seal the test cups (20 mm in diameter and 45 mm in depth), which contained 15 mL of deionized water. The cups were maintained in a controlled incubator (25 °C, 50% RH) for 48 h and weighed. WVP was determined using Equation (4):

(4) $$\mathrm{WVP}=\left(\Delta m\times L\right)/\Delta t\times A\times \Delta p$$

where Δm/Δt (g/s) is the slope of the plot of weight loss versus time, L (m) is the film thickness, A (m^2^) is the area covered by the film, and Δp (Pa) is the water vapor pressure differential across the film under the tested conditions.

### 2.14. Mechanical Properties

The tensile strength and elongation at break of the films were measured by a texture analyzer apparatus (TA plus, Walnut, CA, USA). The samples were cut into 20 mm width and 50 mm length strips. The initial grip separation and the crosshead speed were 30 mm and 30 mm min^−1^, respectively. The tensile strength and elongation at break of the films were calculated based on the following equations [23]:

$$\mathrm{Tensile}\;\mathrm{Strength}\;\left(\mathrm{MPa}\right)=\frac{\mathrm{Fmax}}{W\times T}$$

$$\mathrm{Elongation}\;\mathrm{to}\;\mathrm{break}\;\left(\%\right)=\frac{L-L_{0}}{L_{0}}\times 100$$

where Fmax (N) is the maximum force applied for the strips, *W* is the film width (mm), *T* is the film thickness (m), *L* is the final length (mm), and *L*_0_ is the initial length (mm) of the film sample.

### 2.15. Statistical Analysis

One-way analysis of variance (ANOVA) and Tukey’s multiple tests by IBM SPSS statistics 26.0 and origin 2019 software were used for statistical analyses. The significant level was *p <* 0.05.

## 3. Results and Discussion

### 3.1. Thickness

Table 1 shows the thickness of SPI films before and after storage at different temperatures. It can be seen that the thickness of SPI films had no significant difference (*p* > 0.05) before and after storage at 4 °C and −18 °C for 6 weeks, which were controlled between 0.094 and 0.091 mm. However, it had a small downward trend under the 25 °C storage condition, which was from 0.094 to 0.088 mm, and the downward trend was significant after 4 weeks of storage (*p* < 0.05). The result suggests that high storage temperatures may accelerate the aging process.

### 3.2. Migration and Distribution of Glycerol

#### 3.2.1. Glycerol Migration Rate

**Figure 1:**
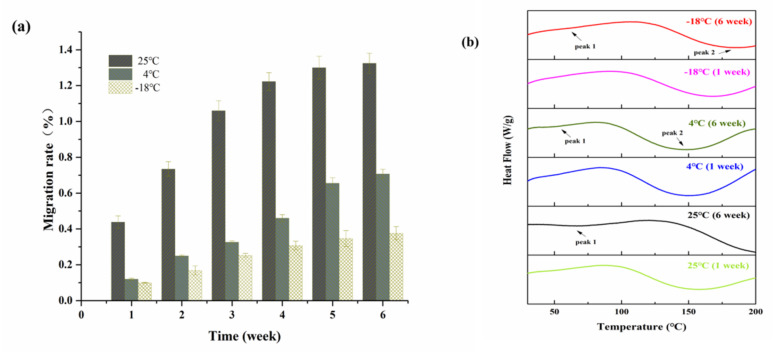
The glycerol migration rate (**a**) and DSC plot (**b**) of SPI edible films stored at different temperatures (25 °C, 4 °C, and −18 °C).

Glycerol migration is an important film property parameter, as the large surface area of the SPI film should not be neglected. Figure 1a shows the glycerol migration rate under different storage temperatures. It can be observed that with the extension of storage time, the glycerol migration rate gradually increased. As a plasticizer, glycerol can increase the mechanical properties of protein films and be well dispersed in it [24,25,26]. However, the migration of glycerol was inevitable due to its low molecular weight. Yan et al. confirmed that soy protein molecules interact with glycerol molecules through hydrogen bonds [15], where the binding limitations and protein types (including polarity, structure, and functional groups) between glycerol and protein molecules are considered the important factors that lead to plasticizer loss and migration [27].

Compared with 4 °C and −18 °C, the glycerol migration rate of SPI films at 25 °C obviously increased and showed a slowly growing trend after 4 weeks of storage (25 °C > 4 °C > −18 °C). It can be seen that storage temperatures had a great influence on the migration content of glycerol, confirming that the glycerol migration state was strongly affected by thermodynamic effects [13]. With the increase in storage temperatures, the movement of water and glycerol molecules accelerated; thus, glycerol migrated to the films’ surface with the water evaporation.

#### 3.2.2. Differential Scanning Calorimetry (DSC)

The DSC results of SPI films stored at different temperatures for 1 and 6 weeks are shown in Figure 1b. It can be seen that the DSC curves of the SPI protein films stored after 1 week had little difference under all storage temperatures, while the peak position did not change by much, but endothermic peaks appeared at temperatures of 150–170 °C. However, when storage time reached 6 weeks, weak endothermic peaks appeared at about 65 °C, 55 °C, and 62 °C at 25 °C, 4 °C and −18 °C, respectively, which may be related to the reduction in weak energy interaction between proteins for denaturation and secondary molecular relaxation [28]. The films at 4 °C and −18 °C showed obvious endothermic peaks at 151 and 185 °C after 6 weeks of storage, respectively, while a significant change can be observed at peak 2 under the storage condition of 25 °C, and the peak at 158 °C in the first week disappeared after 6 weeks of storage.

**Figure 2:**
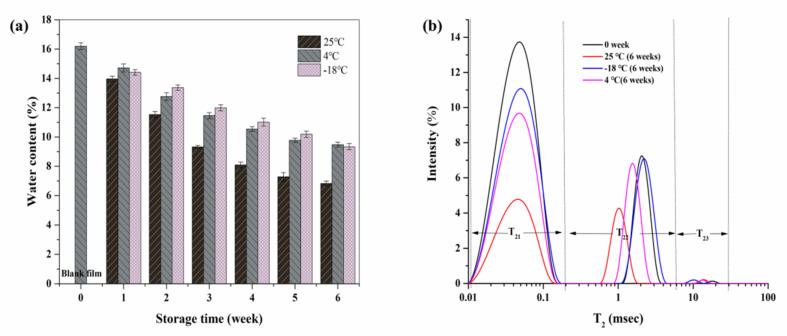
The water content (**a**) and LF-NMR (**b**) of SPI edible films stored at different temperatures (25 °C, 4 °C, and −18 °C).

For the main component of soy protein isolate of globulin, its thermal stability is mainly maintained by hydrogen bonds. The endothermic peak at 150 to 180 °C may be due to the destruction of hydrogen bonds among molecules in the SPI film [29]. Tang et al. considered that the destruction of hydrogen bonds between proteins and plasticizers, which resulted in endothermic peak generation at high temperature, was the key reason to retain the structure of the protein in SPI films [28]. The disappearance of the endothermic peak at high temperature after 6 weeks of storage at 25 °C proved that the glycerol plasticizer and water had largely migrated out, which was consistent with the results of the glycerol migration rate (Figure 1a) and moisture content (Figure 2a) of SPI films. Overall, the changes in thermodynamic properties differing by different storage temperatures was much more related to the reduction in inter- or intra-molecular hydrogen bonds for glycerol migration and water loss.

### 3.3. Migration and Distribution of Water

#### 3.3.1. Water Content and Distribution

It has been reported that water can also be used as an effective plasticizer for films [30]. A change in water content in the film with the influence of storage conditions can be seen from Figure 2a. We can observe that the water content showed a downward trend with the extension of storage time (25 °C > 4 °C > −18 °C), and water loss accelerated and decreased by 9.37% at 25 °C, before the changes slowed and tended to equilibrium after 4 weeks. While the water content decreased by 6.72% slowly after storage at 4 °C, it was the same trend as that at −18 °C (approximately 6.86% less).

LF-NMR was used to determine the water state in SPI films before and after storage (Figure 2b). T_2_ was the binding degree between water and solid matter, and it was confirmed that the greater the leftward peak position, the firmer the binding capacity of water and solid [31]. It can be observed that three peaks appeared in the LF-NMR spectra of the SPI films, and the transverse relaxation times can be divided into three parts: T_21_(0–0.1 ms), T_22_(0.1–1 ms), and T_23_(1–100 ms). T_21_ represented strongly bound water; it is mainly the hydrogen bonds formed by groups on the molecular chain of soybean isolate protein with water molecules. T_22_ represented loosely bound water; it is speculated that this is mainly caused by the strong hydrogen bonds formed between glycerol molecules and water molecules. T_23_ represented free water that binds on the SPI films’ surface and inside with extremely weak bonds. After 6 weeks of storage, the amplitude of bound water and loosely bound water all showed an overall decreasing trend, while free water was less abundant in the SPI films. It had the greatest effect on water distribution after being stored at 25 °C, and it showed decreases by 65% and 42% in the peak signal for bound water and loosely bound water, respectively. This phenomenon indicated that high storage temperatures led to an increase in the mobility of water molecules, and free water decreased obviously. Studies have confirmed that the presence of polyol plasticizers such as sorbitol can form strong hydrogen bonds with water, making the bound water more stable [20]. Thus, it is speculated that the reason for the decrease in the content of strongly bound water and loosely bound water may be related to the decrease in the binding of glycerol and water molecules caused by glycerol migration and water evaporation.

It can be observed that there seemed to be a positive correlation between the change in water content and glycerol migration rate combined with glycerol migration rate (Figure 1a). At 25 °C of storage, the glycerol migration rate and water content showed a gentle trend after 4 weeks, while glycerol migration rate and moisture content showed little change at a −18 °C storage temperature, as we all know that the water evaporation rate is mainly dependent on temperature [32]. The evaporation of water might lead to the aggregation of glycerol molecules that bound with this part of water molecules, which could accelerate glycerol migration to the films’ surface. Ket-On et al. also observed that under controlled relative humidity, plasticizers that were added in the whey protein film were accelerated by the increase in storage temperature [1].

#### 3.3.2. Thermogravimetric Analysis (TGA)

**Figure 3:**
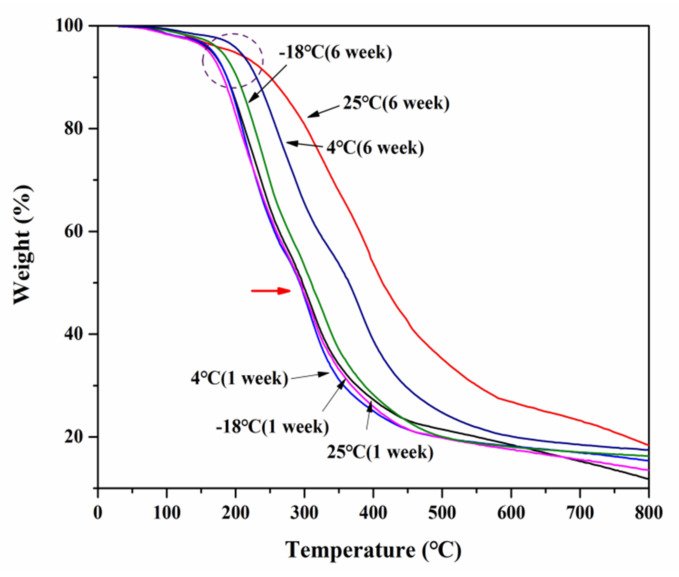
TGA of SPI films at different storage temperatures (25 °C, 4 °C, −18 °C).

TGA was used to observe the impact of storage temperatures on the weight loss profiles that depended on the glycerol migration and water evaporation situation of the SPI films. Figure 3 shows the TGA curves of SPI films stored at 25, 4, and −18 °C after 1 and 6 weeks. The TGA curves of the films did not significantly change in the early stage of storage. The thermal properties were more affected by the moisture content of SPI films and reflected the changes in principal components: protein and plasticizer [2]. A high water content was present in films with a shorter storage time, and a trend of decreasing weight was seen at around 100 °C. We can also find that peaks around 200 °C existed in most SPI films except those stored at 25 °C and −4 °C after 6 weeks, mainly due to weight changes caused by the loss of water with a fraction of glycerol. Koupantsis observed that at 150 and 240 °C, the main weight change was caused by glycerol loss [11]. It proved that the migration of water and glycerol during storage was able to affect the thermodynamic properties of the SPI films. The result is close to the findings of Bagheri et al., who showed that the glycerol loss of alginate films occurred at the maximum temperature around 210 °C [16]. Meanwhile, Gao et al. also detected a loss of glycerol at 218 °C from the TGA curve of the alginate film [33].

### 3.4. Microcosmic State

#### 3.4.1. SEM

**Figure 4:**
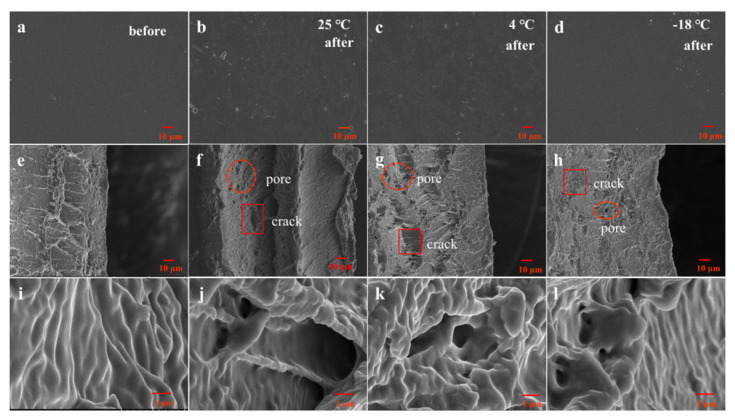
SEM of SPI edible films’ surfaces and sections before and after storage for 6 weeks at different temperatures (25 °C, 4 °C, and −18 °C). Columns from left to right are images of blank SPI films (with no storage) and films after 6 weeks of storage at 25 °C, 4 °C, and −18 °C respectively; Rows from top to bottom, are the surface of SPI films (magnification 1000×), section of SPI films (magnification 1000×), and section enlarged view of SPI films (magnification 15K×), respectively.

SEM was used to observe the surface and cross-sectional structure changes of the SPI film. The results are shown in Figure 4. It can be observed that the surface and cross-section of SPI films changed by varying degrees after 6 weeks of storage, and the changes were especially obvious after storage at 25 °C. The trend is the same as that in the report by Piccirilli [1].

Compared with the blank SPI films (without storage), the surface of the SPI films (Figure 4b–d) revealed the slightly rough surface after storage under all conditions (1000×), while the cross-section changed greatly when observed under 1k× (Figure 4e–h) and 15k× (Figure 4g,i,k,l). It showed that the size of pores in the cross-section of the films increased with the storage temperature. The results are the same as those of the whey protein film microstructure reported by Anker that when using glycerol as the plasticizer, the films presented larger pores [34]. This phenomenon indicated that glycerol as a small molecule was easily affected by storage condition changes [10], which led to a larger gap between SPI molecules, making the originally dense structure porous and fragile. Therefore, it is possible to create pores during storage [11]. Meanwhile, with increasing temperatures, the weak bond strength between glycerol and protein decreased. In addition, the amount of migration increased under the stimulation of external factors, and the number of pores also increased with glycerol migration and the growth in water loss.

At the same time, it can be observed from the microstate of the cross-section that the ductility of the SPI films reduced with the increase in storage temperature, which was the lowest at 25 °C, but it was difficult to observe changes at 4 °C and −18 °C. This suggested that protein aggregation occurred with temperatures increasing significantly in SPI films at the later stage of storage [35], which was the main reason that the structure of SPI films around the pores was more compact (Figure 4f) even though there were more pores in SPI films at 25 °C.

#### 3.4.2. ATR-FTIR

**Figure 5:**
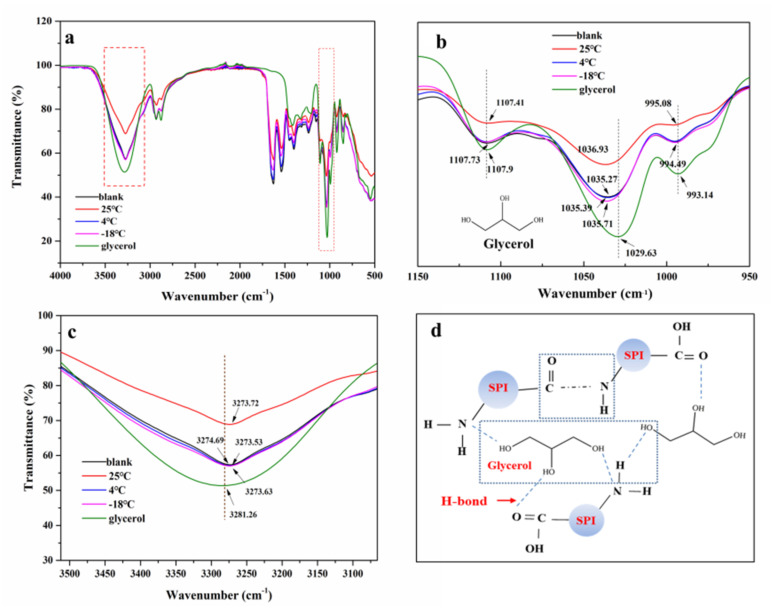
Attenuated total reflection infrared spectroscopy (ATR-FTIR) of SPI edible films before (blank) and after storage by 6 weeks at different temperatures ((**a**) 500–4000 cm^−1^; (**b**) 950–1150 cm^−^^1^; (**c**) 3000–3510 cm^−^^1^; (**d**) Molecular interaction between glycerol and soy protein isolate).

The ATR-FTIR spectrum of pure glycerol and SPI films before (blank) and after storage at different temperatures (6 weeks) are shown in Figure 5. Films after 4 °C and −18 °C storage showed generally similar spectrum features when compared with the blank SPI films, which are consistent with the SPI film infrared spectrum results by Li et al. [36], while differences between SPI spectra of 25 °C compared with 4 °C and −18 °C can be observed. The narrowing of the intensity of the bands at the characteristic peak positions was obtained.

In the region of 800–1150 cm^−1^, we can observe that the main absorption bands of glycerol were represented here. Moreover, the results showed that the peaks at 1107.9 and 1029.63 cm^−1^ may correspond to the Csp3-O bond of glycerol [11]. Compared with blank SPI film, the spectrum band region at 1036.93 and 1107.41 cm^−1^ of the films stored at 25 °C was significantly decreased (Figure 5a). This spectrum region remained with no significant difference after 6 weeks of storage at 4 and −18 °C. The reason for this result was mainly due to the migration of small molecules of the SPI film during storage and the cross-linked aggregation of components within the films. With the migration of glycerol, the characteristic peak area of glycerol molecules in SPI films decreased.

In the range of 3000–3600 cm^−1^, Piccirilli et al. found that the main absorption peak was attributed to the free and bound O-H and N-H groups of protein and water. This region was characterized by a broad absorption band nearly at 3263 cm^−1^ for all proteins [1]. In this study, the glycerol molecule showed a broad absorption band at 3281.26 cm^−1^ in this region (Figure 5c). After 6 weeks of storage, the absorption band of the films at 4 and −18 °C was similar to those the blank films, while this absorption band of the stored film at 25 °C was narrower than that of the blank film, which may relate to the cross-linking of the protein network. The loss of water can reduce the number of free -OH groups that can also lead to the narrower band [1,37].

### 3.5. Physical Properties of SPI Films

#### 3.5.1. Swelling Ratio

The swelling ratio (SW) can directly reflect the absorption and binding ability of the SPI film to water molecules. As shown in Table 2, it can be observed that the swelling ratio of SPI films changed little in the early stage (0–2 weeks) but decreased significantly (*p* < 0.05) after 3 weeks of storage under all storage conditions. Furthermore, at 25 °C, the swelling ratio decreased by 4.04%. However, the swelling ratio decreased by 2.9% and 1.8% at 4 °C and −18 °C, respectively. This might be due to the spatial structure changes caused by protein aggregation and the plasticizer loss of SPI films with storage. Glycerol has a strong ability to absorb water molecules [6], and with the migration of glycerol, the reduction in the glycerol molecules in films correspondingly decreased the attachment ability of water molecules.

#### 3.5.2. Transmittance of Films

**Figure 6:**
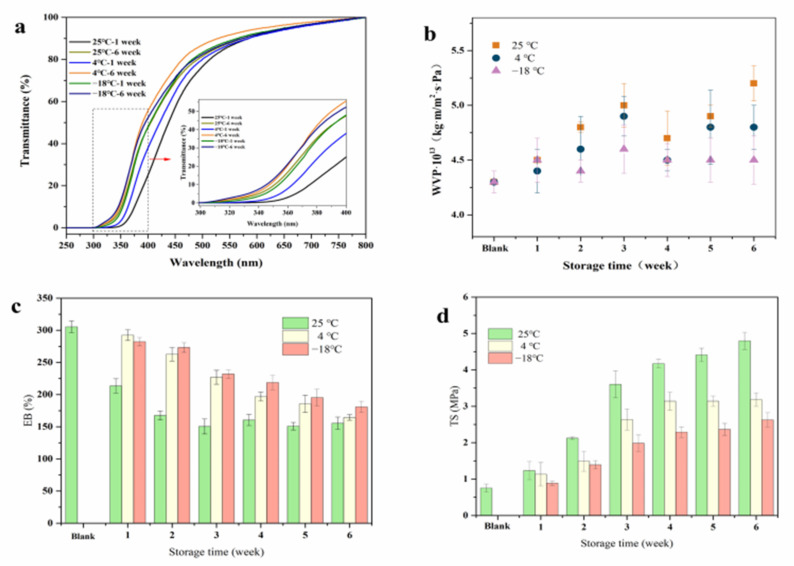
Transmittance (**a**), WVP (**b**), EB (**c**), and TS (**d**) of SPI films after storage for 6 weeks.

As an important index of edible protein film, light transmittance can be affected by storage conditions [38]. Due to the inherent group of protein having the property of absorbing ultraviolet light, it resists the damage of food oxidation and corruption caused by ultraviolet light [39]. The results are shown in Figure 6a. The SPI film was in the range of short-wave ultraviolet and mediumwave ultraviolet (280–100 nm and 315–280 nm). The transmittance increased with the storage time in the long-wavelength ultraviolet region (400–315 nm) under 25 °C conditions, while it was significantly lower than those at 4 °C and −18 °C. Combined with the results of SEM (Figure 4f), the cross-sections of the SPI films were more compact after storage at 25 °C, so the UV absorption capacity enhanced and the light transmittance per unit area of SPI films decreased. This may be due to the aggregation and cross-linking between protein molecules caused by the increase in the contact sites after the migration of glycerol and water molecules.

#### 3.5.3. Water Vapor Permeability (WVP)

The factors that can influence water vapor permeability are film hydrophilicity, moisture flow status, films’ structural composition, etc. [14]. It can be seen from Figure 6b that the WVP showed a similar change trend in general, and the temperature changed most at 25 °C and least at −18 °C, but they roughly showed the same trend of increasing first followed by decreasing, gradually equilibrating after 5 weeks. Due to strong hydrophilicity, a large number of evaporated water molecules could combine with glycerol molecules in the SPI films, resulting in the increase in local water content and the increase in WVP value during the first 3 weeks. The WVP values of SPI films showed an increasing trend with storage temperature because of the acceleration of movement of hydrophilic groups in the SPI films, and these groups all adsorbed water molecules. This faster movement would lead to the faster loss of water molecules, increasing the WVP value of SPI films. Referring to the SEM images (Figure 4f), the pores of the film stored at 25 °C were large, and the presence of loose cracks may be one of the main reasons for the higher WVP values at 25 °C than 4 °C and −18 °C after 6 weeks of storage.

#### 3.5.4. Mechanical Properties

The mechanical properties can directly reflect whether protein films are stable during their shelf life [40]. It mainly includes tensile strength (TS) and elongation at break (EB). Figure 6c,d revealed that the TS of the SPI films showed an overall increasing trend with storage temperature, while the EB showed the opposite trend. The initial TS value enhanced from 0.75 ± 0.11 to 4.8 ± 0.24, 3.18 ± 0.19, and 2.63 ± 0.19, while the EB value reduced by 49.18%, 46.19%, and 40.78% after storage at 25 °C, 4 °C, and −18 °C, respectively. The most obvious changes were observed at 25 °C, and the trend of EB and TS changed mostly in the first three weeks of storage, slowing down in the later period. Ket-On et al. found that plasticizer migration and water loss might be the factors affecting the mechanical properties of protein films [14].

Identical to this result, changes in the mechanical properties in this study showed a trend relative to glycerol migration (Figure 1a) and water loss (Figure 2a). It may be that the mobility of molecules within the SPI films was stronger under higher storage temperature conditions. With the migration of glycerol and the loss of water, the protein intermolecular cross-linking was enhanced, and the weak bonds between plasticizers and soy protein molecules were replaced by stronger bonds; thus, TS increased. Meanwhile, with the loss of glycerol, the plasticization effect was weakened obviously, which made the SPI film less flexible and more rigid, thus reducing EB values. The results are consistent with the observation by Artharn et al., who found that under 28–30 °C storage conditions, TS increased and EB decreased in round scad protein-based films [41]. An increasing TS value of WPC-based films was also observed by Pérez et al. under 25 °C storage conditions [25]. In general, storage temperatures have a close relationship with the mechanical properties of SPI protein films. The TS value can increase, but the EB value may be reduced by storage [42,43].

## 4. Conclusions

In this study, glycerol and water migration were effectively monitored by analyzing the glycerol migration rate and water content, respectively. ATR-FTIR effectively proved glycerol migration changes, NMR effectively showed the water migration situation, and SEM obviously demonstrated microscopic changes in SPI films. This study provides a theoretical basis for improving the quality of edible packaging materials in the future. This research presented the effect of different storage temperatures (25 °C, 4 °C, and −18 °C) on the glycerol and water migration regularities of SPI edible films. The results revealed that the migration of glycerol and water differed by different storage temperatures. Compared with 25 °C and 4 °C, glycerol migration and water loss were weakest and showed less degradation when the SPI edible films were stored at −18 °C, while a higher loss of plasticizers was found after storage at 25 °C and even tended to equilibrate slowly after 4 weeks of storage. After 6 weeks, the mechanical properties, thermodynamic properties, swelling ratio, WVP, and transmittance ratio of the films changed significantly, and the peak areas of free hydroxyl groups and characteristic peaks of glycerol in the ATR-FTIR spectrum reduced, verifying that glycerol and water migration of the SPI films were highest under the 25 °C condition, and internal quality deterioration may occur concomitantly. SEM showed that pores and cracks emerged in the SPI films’ cross-section after 6 weeks under all storage conditions, and with the increase in storage temperatures, cracks and larger pores were obviously observed, which may lead to the deterioration of films’ packaging properties. In conclusion, the rise in storage temperatures accelerated the migration of glycerol and water, and this was the important factor that led to the different changes in the deterioration in the SPI films’ packaging properties.

## Figures and Tables

**Table 1:** Film thickness (mm) of SPI edible films stored at different temperatures (25 °C, 4 °C, and −18 °C).

Thickness							
Storage Time (Weeks)	0	1	2	3	4	5	6
Control 25 °C	0.094 ± 0.002 ^a^	0.094 ± 0.001 ^a^	0.094 ± 0.003 ^a^	0.093 ± 0.000 ^a^	0.092 ± 0.001 ^ab^	0.089 ± 0.001 ^b^	0.088 ± 0.001 ^b^
Control 4 °C	0.094 ± 0.001 ^a^	0.095 ± 0.003 ^a^	0.093 ± 0.002 ^a^	0.094 ± 0.002 ^a^	0.093 ± 0.004 ^a^	0.091 ± 0.002 ^a^	0.093 ± 0.003 ^a^
Control −18 °C	0.094 ± 0.002 ^a^	0.094 ± 0.004 ^a^	0.095 ± 0.02 ^a^	0.093 ± 0.003 ^a^	0.092 ± 0.002 ^a^	0.093 ± 0.001 ^a^	0.094 ± 0.002 ^a^

Values are given as mean ± standard deviation. (a, b) Means in the same row with different superscript letters are significantly different (*p* < 0.05).

**Table 2:** Swelling ratio of SPI edible films stored at different temperatures (25 °C, 4 °C, and −18 °C).

Temperature	Storage Time (Weeks)						
	0	1	2	3	4	5	6
25 °C	16.37 ± 0.15 ^a^	16 ± 0.17 ^a^	15.43 ± 0.15 ^b^	14.97 ± 0.12 ^c^	14.27 ± 0.15 ^d^	13.43 ± 0.21 ^e^	12.33 ± 0.21 ^f^
4 °C	16.37 ± 0.15 ^a^	16.33 ± 0.15 ^a^	15.9 ± 0.17 ^b^	14.90 ± 0.10 ^c^	14.53 ± 0.15 ^d^	13.73 ± 0.15 ^e^	13.47 ± 0.15 ^e^
−18 °C	16.37 ± 0.15 ^a^	16.20 ± 0.10 ^ab^	15.97 ± 0.21 ^b^	15.63 ± 0.12 ^c^	15.27 ± 0.15 ^d^	14.73 ± 0.12 ^e^	14.57 ± 0.15 ^e^

Values are given as mean ± standard deviation. Means in the same row with different superscript letters are significantly different (*p* < 0.05).

## Data Availability

The data presented in this study are as described in the individual figures and tables.
